# Potential of Regular Consumption of Cameroonian Neem (*Azadirachta indica* L.) Oil for Prevention of the 7,12-Dimethylbenz(a)anthracene-Induced Breast Cancer in High-Fat/Sucrose-Fed Wistar Rats

**DOI:** 10.1155/2019/2031460

**Published:** 2019-04-07

**Authors:** Stephane Zingue, Kevine Kamga Silihe, Innocent Fouba Bourfane, Ali Boukar, Alain Brice Tueche, Amstrong Nang Njuh, Dieudonné Njamen

**Affiliations:** ^1^Department of Life and Earth Sciences, Higher Teachers' Training College, University of Maroua, P.O. Box 55, Maroua, Cameroon; ^2^Department of Biochemistry, Faculty of Sciences, University of Yaounde 1, P.O. Box 812, Yaounde, Cameroon; ^3^Department of Biological Sciences, Faculty of Science, University of Maroua, P.O. Box 814, Maroua, Cameroon; ^4^Department of Animal Biology and Physiology, Faculty of Science, University of Yaoundé 1, P.O. Box 812, Yaounde, Cameroon

## Abstract

Neem (*Azadirachta indica*) is a tree from the Meliaceae family native to India, where it is considered as one of the most important plants worldwide. The anticancer effects of neem oil on breast cancer cells have been recently reported; however, its* in vivo* effects have not been studied. This prompted us to investigate the protective effects of neem oil on 7,12-dimethylbenz(a)anthracene (DMBA)-induced breast cancer in high-fat/sucrose-fed Wistar rats. Juvenile female Wistar rats were treated either with neem oil at a dose of 3 mL/kg body weight at 3 different frequencies, 2 times/week (Neem 1), 4 times/week (Neem 2), and every day (Neem 3), or with tamoxifen (3.3 mg/kg body weight), starting 1 week prior to DMBA treatment and lasting 12 weeks. Incidence, burden, volume, and histological analysis of mammary tumors were measured. Further toxicological parameters have been assessed. No tumors were detected in rats from the normal group, while all the rats from the negative control group (100%) developed mammary tumors. The regular consumption of neem oil at a dose of 3 mL/kg (2 or 4 times/week) significantly (p < 0.01) and in a dose-dependent manner reduced tumor incidence (80%), burden [35.78% (2 times/week) and 36.09% (4 times/week)], and weight. Neem consumption protected rats against DMBA-induced breast hyperplasia, with an optimal effect when taken 4 times weekly. Interestingly, all the animals that received a daily dose of 3 mL/kg died at the third week of the experiment. Further, animals that took the neem oil 4 times per week developed hepatotoxicity, evidenced by an increase of liver wet weight, transaminase (ALT and AST) activity, and histological abnormalities in liver. This study brings insight into the use of neem oil, which is greatly appreciated in traditional medicine. In summary, we demonstrated for the first time that the regular consumption of neem oil prevents breast cancer, but its excessive consumption is toxic.

## 1. Introduction

Cancer is a growing health problem affecting millions of people worldwide. In 2012, it was estimated that 32.6 million people were suffering from cancer, and it is predicted that 18.5% of the world's population will likely develop cancer before the age of seventy-five [1]. Worldwide, breast cancer is the second most frequent cancer and the fifth cause of cancer-related mortality [2]. In Cameroon, it remains a significant public health challenge as incidence of breast cancer is higher than the world's average, estimated at 2625 per 100,000 women with a resultant high mortality [3, 4]. Various factors can contribute to the onset of breast cancer including but not limited to age, estrogen, genetic factors, and obesity [5]. This latter is a risk factor for breast cancer, especially after menopause, characterized by preadipocytes proliferation and hypertrophic growth of mature ones leading to an increased number and size of adipocytes [6].

Breast cancer continues to be an enigmatic challenge for researchers and is one of the most threats and a major cause of mortality in women worldwide [1]. Treatment options are limited and expensive, and patients experience many adverse effects. Particularly for Africa, treatment cost is high, and centers for diagnosis and management are limited. These, coupled with drug resistance and systemic toxicity of current chemotherapeutic molecules, pile up the difficulties faced using the different anticancer therapies [7, 8]. Faced with these difficulties, the quest for new anticancer molecules which are readily available, effective, and less expensive becomes a serious issue if these problems have to be overcome. In recent years, certain herbal products and ethnomedicines have drawn keen attention of researchers primarily because of convincing anticancer properties with negligible unpleasant side effects and discomfort to patients [9–11]. These products also have mild side effects on patients after consumption.

Neem (*Azadirachta indica*) is a tropical or subtropical fast-growing evergreen with oblique leaves and stout trunk of timber values with insect-repellant properties [11, 12]. It branches profusely with oblique leaves, and its stout trunk is used for timber. It is resistant to drought and high temperature. It has a variety of common names in the local languages, including Indian lilac in English, Azadirakhta in Persian, Arya-veppu in Malayalam, Margosa and neeb in Arabic, Vaypum in Tamil, Tamar in Burmese, Bevu in Kannada, Kohomba in Sinhala, Pokok semambu (Malaysia), Dogon yaro in some Nigerian languages, Vepu, Vempu, Vepa in Telugu, neem in Hindi and Bangla, and nimba in Sanskrit and Marathi. Though basically used for traditional medicine in the treatment of many diseases and illnesses affecting humans, it is also consumed as a vegetable in some parts of the Asian subcontinent [12]. The anticancer properties of neem have been investigated in detail from several scientific angles. Regular use of neem and its preparations has been found to prevent onset of cancer through multiple mechanisms including suppression of proliferation and growth of cancer cells, cell cycle arrest and apoptosis, interference with growth factor signaling, inhibition of angiogenesis, and decrease of tumor cell invasion and migration [12]. The curative effects of this plant have been attributed to the numerous chemical constituents present in various parts of the plant. These include azadirachtin, gedunin, nimbidin, nimbidol, nimbin, salannin, and quercetin. Many parts of neem such as stem, leaves, flowers, fruits, and seeds have demonstrated anticancer effects; however, such effects differ greatly depending on factors such as soil and climate as well as the extraction process. Recently, Sharma et al. [13] demonstrated the cytotoxic effects of neem oil on positive estrogen receptor-ER+ (MCF-7) and ER– (MDA-MB-231) breast cancer cells with CC_50_ of 10 *μ*l/ml and 20 *μ*l/ml, respectively. According to the same authors, the neem oil induced apoptosis and cell cycle arrest at G0/G1 phase in both cell types. However, the* in vivo* effects of neem oil have not been studied, although it is being used increasingly in Cameroonian traditional system. We therefore analyze the* in vivo* anti-mammary cancer effects of neem oil.

## 2. Materials and Methods

### 2.1. Chemicals

The 7,12-dimethylbenz(a)anthracene (DMBA) (purity ≥ 95%) was purchased from Sigma-Aldrich (Stanford, Germany). Tamoxifen citrate (Mylan®) was purchased from MYLAN SAS (Saint-Priest, France).

### 2.2. Plant Material

#### 2.2.1. Collection and Authentication

The seeds of neem (*Azadirachta indica* L.) were harvested in Maroua (Far North Region, Cameroon) in June 2017 around 11:30 a.m. The plant was localized at the geographical coordinates of N10° 35.487' East and E0 14° 18.910' altitude with a “ESTREX” global positioning system. The city of Maroua is in the Sudano-Sahelian agroecological zone, characterized by two seasons: wet (June to September) and dry (October to May). Annual rainfall ranges between 800 and 1000 mm. Annual mean temperature is 298 C, with a maximum of 39 8C in March and minimum of 17 8C in January. This botanical sample was identified and authenticated by M. Victor Nana, a taxonomist at the National Herbarium of Cameroon (HNC-IRA), by comparison to the specimens deposited under the voucher number 4447 SRFK.

#### 2.2.2. Preparation of the Neem Oil

Neem fruit was peeled, cleaned for almonds, and dried in a shade for two weeks. After drying, it was crushed with an iron mortar to make it powder. Then, 2000 g of this powder was mixed with 250 ml of tap water; afterward, the resulting pulp was manually pressed to separate the dough oil by pressing the dough. The supernatant (375 ml) was collected in a clean plastic flask after filtering with a Whatman N°2 filter paper constituting our neem oil.

### 2.3. Animals

Fifty prepubertal female Wistar rats, aged 35-40 days at the beginning of experiment and weighing around 45-65 g, were supplied by the breeding facility of the Laboratory of Animal Physiology, University of Yaounde I (Yaounde, Cameroon). These rats were housed in plastic cages in groups of five at room temperature. The animals had free access to a standard (SD) or high-fat high-sucrose (HFHS) chow (Table 1) and water.

### 2.4. Ethical Consideration

Housing of animals and all experiments were approved by the Cameroon Institutional National Ethic Committee, which adopted all procedures recommended by the European Union on the protection of animals used for scientific purposes (CEE Council 86/ 609; Reg. no. FWA-IRD 0001954).

### 2.5. DMBA-Induced Breast Tumor in High-Fat/Sucrose-Fed Wistar Rats

Thirty-five (35) female rats were acclimatized for 10 days and randomly assigned at age of 50-55 days into 7 groups of 5 animals each. At the beginning of the experiment, animals were divided into two groups: rats fed with standard (n = 5) and high-fat/sucrose (n = 30) diet. Animals received the following treatment schedule: the rats fed with standard diet (SD) served as normal control 1 (NOR-1) and received the vehicle (cotton oil) throughout the experiment. Regarding animals fed with HFHS, group 1 served as normal control 2 (NOR-2) and received the vehicle (cotton oil); group 2 served as negative control (DMBA) and also received the vehicle; group 3 (positive control) received tamoxifen (Mylan®) at the dose of 3.3 mg/kg dissolved in cotton oil. The remaining groups were treated with neem oil at a dose 3 mL/kg (extrapolated from the traditional use) at 3 different frequencies: 2 times/week (Neem 1), 4 times/week (Neem 2), and every day (Neem 3). All treatments were administered via intragastric gavage one week before DMBA administration, once a day around 2:00 p.m., and lasted for 3 months. Breast tumors were induced in group 2 to group 6 of animals that received HFHS by a single subcutaneous dose of DMBA (50 mg/kg) dissolved in 1 ml of cotton oil, while normal group (NOR-1 and NOR-2) received cotton oil only. Experimental rats were weighed weekly and palpated twice a week to check the development of mammary tumors from the first day of acclimatization until the end of the experiment. Tumorous latency (time of tumor appearance) was recorded. On days 1 and 84 of treatment, fasting glycaemia was measured using whole capillary blood obtained from the tip of the tail. The sacrifice and autopsy of either animals that died during the experiment or those that became moribund were carried out. At the end of the 3-month treatment, all survivors were sacrificed after a 12 h overnight nonhydric fasting under valium and ketamine anesthesia (respectively, 10 and 50 mg/kg BW;* i.p.*). On the one hand, blood samples were collected in anticoagulant (EDTA) tubes for hematological analysis; on the other hand, they were collected in dried tubes and centrifuged at 600 × g for 15 min at 4°C for biochemical analysis. Furthermore, the skin was dissected out to expose mammary tumors and all tumors were removed, counted, and weighed. A 1 mm precision sliding caliper (IGAGING®) was used to measure the size of tumors. Afterward, the formula from Faustino-Rocha et al. [14] (length × weight × height ×  *π*/6) was used to calculate the tumor volume. Estrogen target organs were also removed and weighed. Finally, all these organs were fixed in 10% neutral formalin solution for histomorphology.

### 2.6. Histological Analysis

Mammary glands, uterus, vagina, kidneys, and liver were dehydrated by a series of ethanol solution, embedded in paraffin blocks before cutting into 5 *μ*m sections, and stained with hematoxylin and eosin as previously reported by Zingue et al. [9, 15]. Histomorphological changes were determined under an Axioskop 40 microscope connected to a computer where the image was transferred using MRGrab1.0 and Axio Vision 3.1 software (Zeiss Hallbergmoos, Germany). Atlas and histologic classification of tumors of rat mammary gland from Russo and Russo [16] were used in this study.

### 2.7. Biochemical and Hematological Analysis

Regarding biochemical analysis, aspartate transaminase (AST) and alanine transaminase (ALT) activities were measured as previously reported [9, 15] using reagent kits from Fortress Diagnostics Limited (Muckamore, United Kingdom). Capillary glucose measurements were done on days 1 and 84 of the experiment. The quantitative determination of blood glucose was done by the glucose dehydrogenase method, using a specific spectrometer-analyzer OneTouch (OneTouch®, Lille, France).

Different hematological parameters were evaluated using a Mindray BC-2800 Auto Hematology Analyzer from Shenzhen Mindray Bio-Medical Electronics Co., Ltd. These are white blood cell (WBC) count, lymphocytes, monocytes, granulocytes, red blood cell (RBC) count, hematocrit, hemoglobin, mean corpuscular volume (MCV), mean corpuscular hemoglobin (MCH), mean corpuscular hemoglobin concentration (MCHC), and platelets.

### 2.8. Statistical Analysis

Results are presented as means ± standard deviation (SEM) for each experimental group. Statistical analysis of data with GraphPad Prism software version 6.00 (GPW6-242831-RBMZ-03274) was performed using the one-way analysis of variance (ANOVA) followed by Dunnett's post hoc test for multiple comparisons. Statistical significance of differences was considered at a p value < 0.05.

## 3. Results

### 3.1. Effects on Body Weight

**Figure 1:**
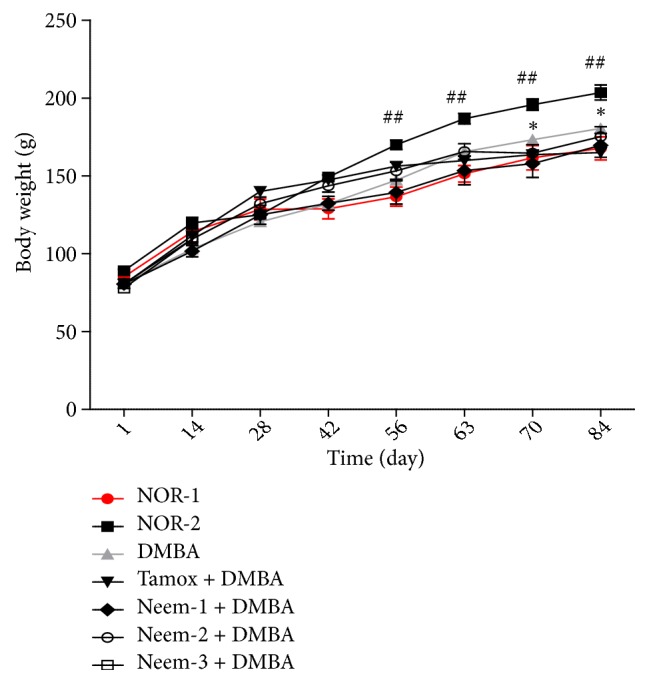
Effects of neem (*Azadirachta indica*) oil on the body weight change in rats treated for 12 weeks. NOR-1: normal rats fed with SD treated with cotton oil; NOR-2: normal rats fed with HFHS treated with cotton oil; DMBA: negative control treated with cotton oil; TAM + DMBA: animals treated with tamoxifen (3.3 mg/kg); Neem + DMBA: animals treated with neem (*Azadirachta indica*) oil at a dose of 3 mL/kg during three different frequencies. All groups except the normal groups (NOR-1 and NOR-2) received subcutaneous dose of DMBA at the dose of 50 mg/kg. Data are represented as mean ± SEM (n = 5). ##p < 0.01 as compared to normal control group 1 (NOR-1). *∗*p < 0.01 as compared to DMBA group.

The body weight change of animals during the 12 weeks of experiment is represented in Figure 1. A significant (p < 0.01) increase of the body weight mass of normal animals fed with HFHS (NOR-2) as compared with normal animals fed with SD (NOR-1) was observed from day 56 until the end of the experiment. No significant change was found between the different rats fed with HFHS, except for animals treated with tamoxifen that presented a significant (p < 0.05) lower body weight as compared to the normal group (NOR-2).

It is important to notice that all the animals treated with a daily dose of 3 mL/kg of neem oil died before the 3^rd^ week of the experiment.

### 3.2. Effects on Glycaemia

**Figure 2:**
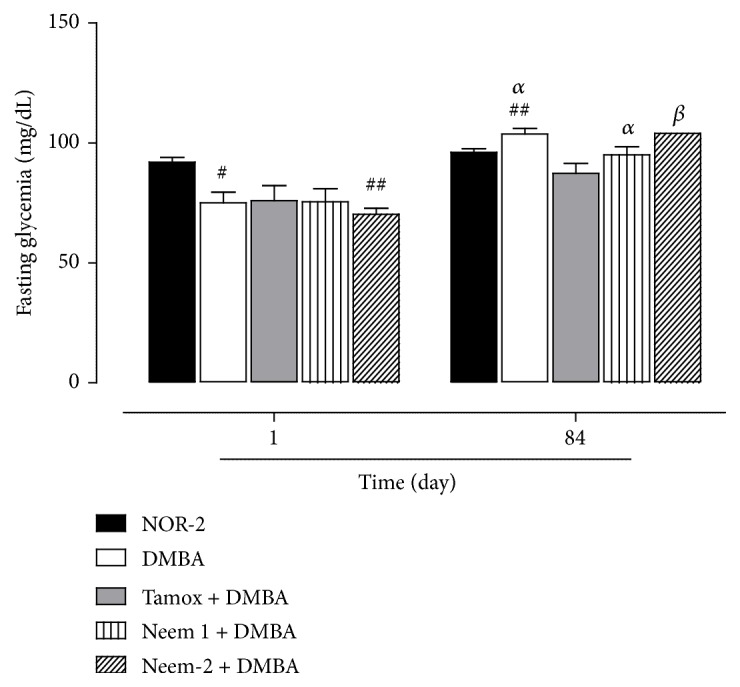
Effects of neem (*Azadirachta indica*) oil on fasting glycemia in rats treated for 12 weeks. NOR-1: normal rats fed with SD treated with cotton oil; NOR-2: normal rats fed with HFHS treated with cotton oil; DMBA: negative control treated with cotton oil; TAM + DMBA: animals treated with tamoxifen (3.3 mg/kg); Neem + DMBA: animals treated with neem (*Azadirachta indica*) oil at a dose of 3 mL/kg during three different frequencies. All groups except the normal groups (NOR-1 and NOR-2) received subcutaneous dose of DMBA at the dose of 50 mg/kg. Data are represented as mean ± SEM (n = 5). #p < 0.05, ##p < 0.01 as compared to normal rats (NOR-2). (*α*): p < 0,05; (*β*): p < 0,01 as compared with themselves at day 1.

Figure 2 shows the blood glucose level taken at the beginning and at the end of the manipulation. A decrease of glycaemia was observed the first day of the experiment with the rats which received DMBA as compared to normal rats (NOR-2), although it was significant only in the negative control (DMBA) and Neem 2 groups. On day 84 of experiment, it was noticed that there is a significant increase (p < 0.01) of the fasting glycaemia in animals of DMBA group as compared to the normal rats. Moreover, the fasting glycaemia of animals belonging to DMBA, Neem 1, and Neem 2 was significantly increased as compared to their own value at the beginning of the experiment. Animals that received tamoxifen prevented this increase of fasting glycaemia.

### 3.3. Effects on Water and Food Intake

**Figure 3:**
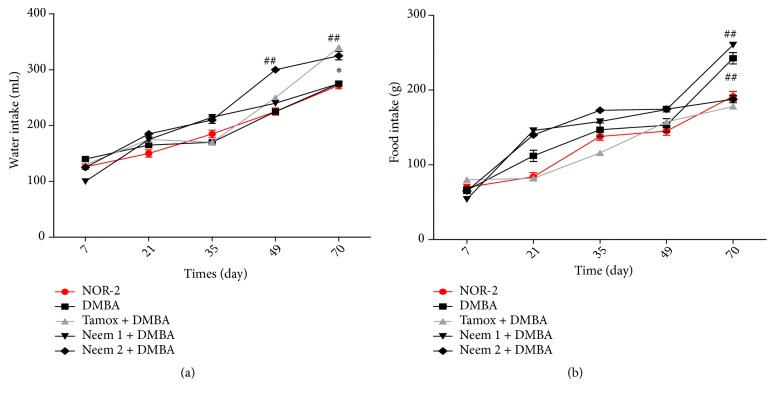
Effects of neem (*Azadirachta indica*) oil on water (a) and food (b) intakes in rats treated for 12 weeks. NOR-1: normal rats fed with SD treated with cotton oil; NOR-2: normal rats fed with HFHS treated with cotton oil; DMBA: negative control treated with cotton oil; TAM + DMBA: animals treated with tamoxifen (3.3 mg/kg); Neem + DMBA: animals treated with neem (*Azadirachta indica*) oil at a dose of 3 mL/kg during three different frequencies. All groups except the normal groups (NOR-1 and NOR-2) received subcutaneous dose of DMBA at the dose of 50 mg/kg. Data are represented as mean ± SEM (n = 5). ##p < 0.01 as compared to normal rats (NOR-2); *∗*p < 0.05 as compared to DMBA group.

Figure 3(a) shows the changes in water intake after 12 weeks of treatment. All the animals showed an increase in water intake throughout the experiment. A significant increase in water intake was observed in animals treated with neem oil four times per week. Moreover, tamoxifen-treated animals also show a significant (p<0.05) increase of water intake as compared to normal animals (NOR-2) at the 35^th^ day of treatment.

Figure 3(b) shows the evolution of food intake in animals during the experiment. A significant increase of food intake was observed in Neem-1 and DMBA groups as compared to the normal group on day 65. In fact, all animals increased food intake during the treatment period, with the exception of the Neem- 2 group which exhibited a stable food intake from the 35th day of treatment.

### 3.4. Effects on Mammary Tumors

Table 2 shows that the animals of the normal group did not develop any tumors throughout the experiment. However, all the rats of the DMBA (n = 5) and Neem 1 (n = 5) groups developed tumors with an average mass of 545.34 ± 43.11 mg/kg and 355.25±16.07 mg/kg, respectively. As expected tamoxifen significantly (p <0.001) protected animals from the incidence of mammary tumors following DMBA administration, with 62.73% of tumor incidence and 203.26 ± 14.21 mg/kg of average tumor weight as compared to the DMBA group. Interestingly, animals treated with neem oil 4 times/week also showed a protective effect, with 80% of tumor incidence and an average tumor weight of 348.84 ± 53.42 mg/kg (p <0.01).

**Figure 4:**
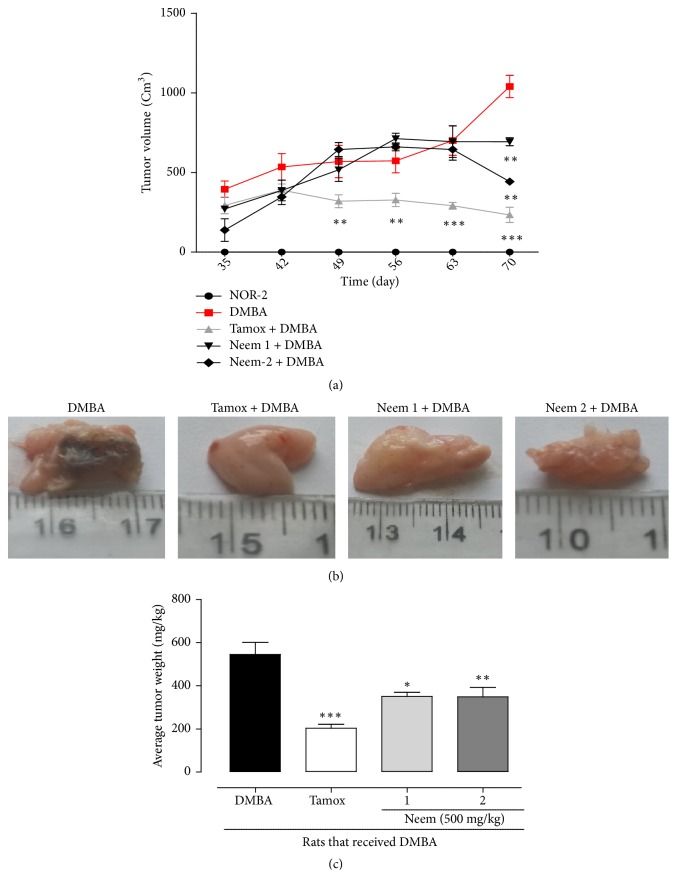
Effects of neem (*Azadirachta indica*) oil on tumor volume (a), tumor morphology (b), and tumor weight (c) of rats treated for 12 weeks. NOR-1: normal rats fed with SD treated with cotton oil; NOR-2: normal rats fed with HFHS treated with cotton oil; DMBA: negative control treated with cotton oil; TAM + DMBA: animals treated with tamoxifen (3.3 mg/kg); Neem + DMBA: animals treated with neem (*Azadirachta indica*) oil at a dose of 3 mL/kg at three different frequencies. All groups except the normal groups (NOR-1 and NOR-2) received subcutaneous dose of DMBA at the dose of 50 mg/kg. Data are represented as mean ± SEM (n = 5). *∗*p < 0.05, *∗∗*p < 0.01, *∗∗∗*p < 0.001 as compared to DMBA.

Figure 4(a) shows the effects of neem oil on tumor volume and weight. The first tumors were observed on day 35 at least in one animal of each group. The average tumor volume of animals from DMBA, Neem 1, and Neem 2 grew continually until day 63. While the tumor volume of DMBA group continued to grow up to 1000 cm^3^, those of Neem 1 (with a mean volume about 700 cm^3^) and Neem 2 (with a mean volume about 300 cm^3^) decreased. Interestingly, the animals that received tamoxifen showed a significant (p < 0.001) lower tumor volume from day 49 until the end of the experiment with an average tumor volume of 150 cm^3^ as compared to DMBA group.

In this study, tamoxifen significantly (p < 0.001) decreased average tumor weight as compared to DMBA control group (Figure 4(b)). The neem oil in a dose-dependent manner significantly (p< 0.01) decreased the average tumor weight as compared to DMBA animals. The graphical representation of the average tumor weight (Figure 4(b)) shows that tamoxifen decreased it by 62% (from 545.34±43.11 to 203.26±14.21 mg/kg BW, P < 0.001), which is the expected activity of tamoxifen. Meanwhile, after the treatment with neem oil, the average tumor weight decreased at the tested dose (500 mg/kg) by a maximum of 36.03% in animal that received neem oil 4 times/week (from 545.34±43.11 to 348.84±53.42 mg/kg).

### 3.5. Effects on Organ Relative Weight

Table 3 depicts the effects of neem oil on the relative weight of different organs after 12 weeks of treatment. There was a significant decrease in the relative weight of uterus (p <0.05), liver (p <0.01), lungs (p < 0.05), and ovaries (p < 0.001) in animals that received tamoxifen as compared to those of DMBA group. The animals treated with neem oil 2 times/week showed a significant (p < 0.05) decrease in the adrenal and ovaries wet weights, while it increased the brain weight as compared to the DMBA animals. Further, animals treated with neem oil 4 times/week presented a significant (p < 0.001) increase of the liver and lungs weight, while it decreased the relative weight of adrenals.

The tamoxifen significantly (p < 0.01) decreased the endometrium height but did not induce significant change in the vaginal epithelial height. The regular consumption of neem oil induced a significant decrease in endometrium height (p < 0.001) as well as the vaginal epithelial height (p<0.05 with Neem 1 and p<0.001 with Neem 2).

### 3.6. Effects on Biochemical and Hematological Parameters

Table 4 depicts the effects of neem oil on some biochemical and hematological parameters. Rats that received neem oil 4 times/week presented a significant (p < 0.001) increase in the ALT (p < 0.05) and AST (p < 0.001) activities as compared to normal (NOR-2) and negative control (DMBA) rats.

No statistical significance was observed between normal and DMBA groups in all measured parameters except for a significant (p < 0.001) increase in red blood count (RBC) and platelets level in the DMBA as compared to normal group (Table 4). Tamoxifen-treated animals showed a significant (p < 0.05) increase in HCBC as compared to DMBA group. Animals treated with neem oil at all frequencies significantly (p < 0.001) prevented the increase of platelet level observed in the DMBA group.

### 3.7. Histomorphological Analysis of Some Organs

**Figure 5:**
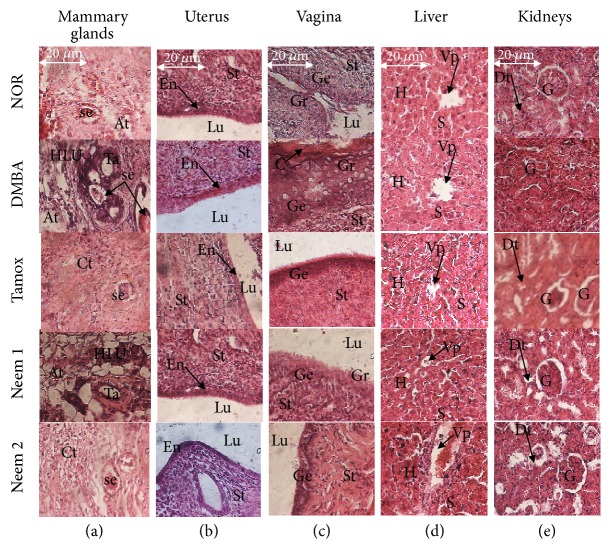
Effects of neem (*Azadirachta indica*) oil on microphotographs H&E 400× of mammary glands (a), uterine (b), vagina (c), liver (d), and kidneys (e) after 12 weeks of treatment. NOR-1: normal rats fed with SD treated with cotton oil; NOR-2: normal rats fed with HFHS treated with cotton oil; DMBA: negative control treated with cotton oil; TAM + DMBA: animals treated with tamoxifen (3.3 mg/kg); Neem + DMBA: animals treated with neem (*Azadirachta indica*) oil at a dose of 3 mL/kg during three different frequencies. All groups except the normal groups (NOR-1 and NOR-2) received subcutaneous dose of DMBA at the dose of 50 mg/kg. Data are represented as mean ± SEM (n =5). La: lumen of alveoli; At: adipose tissue; Se: eosinophil secretion, L: lobular; HLU: hyperplastic lobular unit; St: stroma, En: endometrium, Lu: lumen of uterine; Vp: portal vein, H: hepatocyte, S: sinusoids; Dt: distal tube, G: glomerulus; Ge: stratum germinativum, Gr: stratum granulosum, C: stratum corneum.

The mammary gland microarchitectures of rats from normal group showed normal acini surrounded by a small amount of fibrous conjunctive tissue (Figure 5). Rats that received only DMBA presented mammary gland carcinoma, evidenced by a diminution of the conjunctive tissue as well as a severe hyperplasia of mammary lobules and a dilated ducts filled with tumoral cells. Further, animals treated with neem oil presented a quasi-normal histoarchitecture of mammary glands with low cellular proliferation and low ductal dilation.

On the other hand, histological abnormalities such as sinusoid dilatation, vascular congestion, and inflammation were observed in the liver of animals that took neem oil 4 times/week, as compared to the liver of normal animals. No significant alteration was observed in the microarchitecture of kidneys.

## 4. Discussion

Despite the improvement in the diagnosis and treatment of breast cancer, it remains a major health issue for women [17]. Existing therapies are limited due to substantial adverse effects; therefore, search for novel agents with less or no side effects is warranted in this area. Recently, great interest has been raised in developing natural molecules as potential anticancer agents [18, 19]. In this view, various parts of the neem tree (*Azadirachta indica*) have been shown to inhibit the growth of cancer cells through several mechanisms [20]. The present study deals with the assessment of the* in vivo* antitumor effects of neem oil, based on its reported anticancer effects on breast cancer cells [13]. It was found in this study that neem oil taken 2 times/week and 4 times/week prevents breast tumor incidence and burden, evidenced by the decrease of the number of tumors by animal as well as the tumor volume and weight as compared to the negative control (DMBA). These effects corroborate those of Sharma et al. [13], which demonstrated that neem oil has cytotoxic effects on breast cancer cells. They found that it induced its cytotoxic effects on MCF-7 and MDA-MB-231 cells by G1 phase arrest and by triggering apoptosis through the activation of ROS-mediated mitochondrial dysfunction pathway. The authors attributed these effects to the presence of azadirachtin and a range of limonoids, which are known to stimulate cancer cell death by different cell-death mechanisms including the intrinsic pathway of apoptosis [21]. Nimbolide induces apoptosis in MCF-7 and MDA-MB-231 human breast cancer cells via extrinsic and intrinsic pathways [22]. These compounds and their described mechanisms could account for the* in vivo* anticancer effects of neem oil observed in this study. Preclinical studies have shown compelling evidence suggesting that the anticancer effects of neem are mediated through modulation of multiple cellular processes. Numerous bioactive compounds have been isolated from neem oil and could explain the chemopreventive and anticancer therapeutic efficacy of* A. indica*. There are essentially triterpenoids and steroids: nimbin, nimbinin, nimbidic acid, salannin, meliantriol, azadirone, epoxyazadiradione, azadiradione, gedunin, 7-deacetyl-gedunin, meldenin, salannin-lactone, nimbin-lactone, vepinin, nimbidinin, nimbinene, nimbandiol, 3-deacetylsalannin, salannol, 6-o-acetyl-nimbandiol, 1,3-diacetyl-vilasinin, and 6-deacetyl nimbinene [12]. In fact, neem components inhibit cell proliferation, free radical scavenging; induce cell death including apoptosis and autophagy, immune surveillance, anti-inflammatory effects, antiangiogenic effects; reduce cellular oxidative stress; and prevent invasion and metastasis [12]. It is probable that neem employs the pleiotropic effects of various phytoconstituents to produce its broad chemopreventive and anticancer activities.

Given that neem-based preparations have been consumed for several millennia, the normal consumption of neem products can be regarded as absolutely safe [23]. It is assumed that the consumption of Neem products is absolutely safe since its preparations have been consumed over several generations. Neem seed oil was found to be nontoxic to rats when given via oral route. When administered through the oral route, neem oil was found to be nontoxic. However, when given intravenously or intraperitoneally, it caused death in rats with 24 h LD50 values of 14 mL/kg [23]. It however caused death in rats when administered intravenously or intraperitoneally with 24 h LD50 values of 14 mL/kg. In this study, a toxic effect was found with an excessive consumption of neem oil. In fact, animals that received a dose of 3 mL/kg of neem oil every day died before the third week of the experiment. In addition, animals that received neem oil 4 times/week presented a significant increase in the weights of liver and lung which is an indication of toxic effects of tested substances [24]. Moreover the activities of transaminases (ALT and AST) were also significantly increased. Of note, alanine transaminase (ALT), specific to hepatocytes and aspartate transaminase (AST), found in liver, cardiac muscle, and kidney [25, 26], is well-known as a marker of cell damage, especially hepatocyte necrosis [27]. The transaminases are generally known to be markers of cell damage particularly hepatic damage. However their distribution throughout the body vary, with alanine aminotransferase (ALT) specific to hepatocytes and aspartate aminotransferase (AST) found in liver, cardiac muscle, and kidney. Generally, any damage of the parenchymal liver cells results in elevation of both transaminases in the blood and can be taken as a first sign of cell damage that leads to the outflow of the enzymes into the serum [28]. An elevation in both enzyme activities is generally interpreted as damage of the parenchymal liver cells, resulting in an overflow of these enzymes into serum. The use of neem or its products may also be a reason for damage to liver and kidney that may result in jaundice and in low or no urine production, respectively. A jaundice and low or no urine production may come as a result of liver and kidney damage, a consequence of consuming neem or its products. To verify the latter assumption, we assessed the microarchitecture of liver. The histopathological examinations of the liver of animals belonging to all groups except those of Neem 2 group showed normal integrity of these tissues. However, the increase of transaminases in the present study was also accompanied by histological abnormalities such as sinusoid dilatation, vascular congestion, and inflammation in the liver. The aforementioned observations strengthen our hypothesis on the toxicity due to excessive consumption of neem oil. As far as hematological parameters are concerned, a significant decrease of red blood cell was noted in all animals that received the DMBA. Furthermore, animals that received neem oil 4 times/week presented more severe anemia. These observations are in line with those of Haque et al. [29] who argued that although neem has some hematostimulatory effect in murine, it may also destroy red blood corpuscles.

## 5. Conclusion

In summary, the regular consumption of neem oil at a dose of 3 mL/kg (2 or 4 times/week) significantly reduced tumor incidence (80%), burden, and mass in a dose-dependent manner. Neem consumption protected rats against DMBA-induced breast hyperplasia. However all the animals that received a daily dose of 3 mL/kg died at the third week of the experiment. Further, animals which took neem oil 4 times a week presented a hepatotoxicity. This study brings new insight into the use of neem oil, which is greatly appreciated in traditional medicine. In line with the previous* in vitro* anticancer effects reported, we demonstrated for the first time that the regular consumption of neem oil prevents breast cancer in rats, but its excessive consumption is toxic.

## Figures and Tables

**Table 1:** Rat chow composition (g/kg).

Ingredients	*Standard diet *	*High fat/sucrose diet *
Milk	/	145
Sucrose	/	290
Wheat flour	216	188
Corn flour	324	237
Margarine	/	143.3
Palm oil	50	55
Cellulose	/	30
Mineral salts	20	20
Vitamins	5	5
Water	385	/

**Table 2:** Effects of neem (*Azadirachta indica*) oil on some tumor parameters of rats treated for 12 weeks.

Items	NOR	DMBA	Tamox + DMBA	Neem 1 + DMBA	Neem 2 + DMBA
Number of rats with tumors/total rats	0/5	5/5	3/5	5/5	4/5
Tumor incidence (%)	0	100 ^###^	60*∗∗∗*	100	80*∗∗*
Average tumors weight (mg/kg)	-	545.34±43.11	203.26±14.21*∗∗∗*	355.25±16.07*∗*	348.84±53.42*∗∗*
% inhibition related to tumor weight	-	-	62.73	34.86	36.03
Total tumor burden (g)	0	3.27	1.21	2.10	2.09
% inhibition related to tumor burden	-	-	62.99	35.78	36.09
Tumor volume (Cm^3^)	-	701.01± 104.25	289.96±118.91*∗∗∗*	223.09± 62.52*∗∗∗*	139.77±30.83*∗∗∗*

NOR-1: normal rats fed with SD treated with cotton oil; NOR-2: normal rats fed with HFHS treated with cotton oil; DMBA: negative control treated with cotton oil; TAM + DMBA: animals treated with tamoxifen (3.3 mg/kg); Neem + DMBA: animals treated with neem (*Azadirachta indica*) oil at a dose of 3 mL/kg at three different frequencies. All groups except the normal groups (NOR-1 and NOR-2) received subcutaneous dose of DMBA at the dose of 50 mg/kg. Data are represented as mean ± SEM (n = 5). ###p < 0.001 as compared to normal rats (NOR-2); *∗*p < 0.05, *∗∗*p < 0.01, *∗∗∗*p < 0.001 as compared to DMBA.

**Table 3:** Effects of neem (*Azadirachta indica*) oil on organs weight of rats treated for 12 weeks.

Organs	NOR	DMBA	Tamox + DMBA	Neem 1 + DMBA	Neem 2 + DMBA
*Estrogen target organs*					
Uterus	1469.48± 102.53	1582.36± 93.59	819.54± 77.55*∗*	1577.11± 158.96	1981.88± 141.37
Ovaries	723.19± 72.11	882.51± 44.50	409.33± 14.03*∗∗∗*	683.52± 32.22*∗*	836.13± 16.01
Endometrium height	14.58± 0.22	13.30± 2.72	7.52± 1.75*∗∗*	5.76± 0.21*∗∗∗*	4.60± 0.01*∗∗∗*
Vaginal height	24.34± 0.83	20.34± 0.50	18.34± 0.03	13.51± 0.52*∗*	8.51± 1.02*∗∗∗*

*Other organs*					
Liver	18544.58± 550.16	17195.25± 462.30	15956.35±230.36*∗∗*	18047.65± 478.10	20497.02± 313.70*∗∗∗*
Lungs	5507.55±275.62	4296.46± 170.08	4078.32±133.02 *∗*	4377.30± 258.86	5988.14± 223.65*∗∗∗*
Spleen	1606.99± 57.93	1221.45± 52.61#	1442.81± 76.46	1450.50± 91.55	1432.21± 68.33
Adrenals	392.50± 32.89	620.63± 22.76##	510.04± 23.65	453.95± 62.17*∗*	314.61± 13.52*∗∗∗*
Kidneys	3508.11± 90.54	3574.44±81.16	3621.88± 65.18	3800.66± 175.32	3812.29±102.00
Femur	1676.57± 50.72	1581.92± 36.14	1399.61± 75.42	1523.45±105.61	1492.18± 42.24
Brain	5893.52± 75.05	6623.14± 121.70	6854.20± 223.93	7579.03± 122.88*∗*	6713.79± 342.75

NOR-1: normal rats fed with SD treated with cotton oil; NOR-2: normal rats fed with HFHS treated with cotton oil; DMBA: negative control treated with cotton oil; TAM + DMBA: animals treated with tamoxifen (3.3 mg/kg); Neem + DMBA: animals treated with neem (*Azadirachta indica*) oil at a dose of 3 mL/kg at three different frequencies. All groups except the normal groups (NOR-1 and NOR-2) received subcutaneous dose of DMBA at the dose of 50 mg/kg. Data are represented as mean ± SEM (n = 5). #p < 0.05, ##p < 0.01 as compared to normal rats (NOR-2); *∗*p < 0.05, *∗∗*p < 0.01, *∗∗∗*p < 0.001 as compared to DMBA.

**Table 4:** Effects of neem (*Azadirachta indica*) oil on some biochemical and hematological parameters.

Items	NOR	DMBA	Tamox + DMBA	Neem 1 + DMBA	Neem 2 + DMBA
*Transaminases*					
ALT	63.5± 2.37	69.33± 2.86	65.2± 2.35	55.16± 3.28	79.1± 1.21*∗*
AST	45.5± 0.79	47.33± 1.28	49.6± 4.91	42.5± 1.85	61.2± 0.71*∗∗∗*

*Hematological parameter*					
WBC (×10^3^*μ*L^−1^)	13.3± 0.66	8.33± 0.54	10.77± 1.67	8.37± 1.67	5.5± 0.17
Lymphocytes (%)	43.2± 0.66	50.43± 1.43	50.775± 2.33	56.45± 1.69	53.6± 0.45
Monocytes (%)	18.95± 0.67	19.4± 1.52	18.875± 1.41	18.075± 1.13	18.3± 0.28
Granulocytes (%)	37.85±1.34	30.16± 0.94	24.225±1.53	25.475± 2.21	27.85±0.44
RBC (×10^3^*μ*L^−1^)	5.57± 0.16	1.40± 0.03###	1.21± 0.14	1.395 ± 0.07	1.07± 0.09*∗∗*
Hematocrit (%)	7.58± 0.66	7.36± 0.13	6.95± 0.93	6.55± 0.68	6.1± 0.28
MCV (fL)	173.7± 19.37	102.23± 2.42#	147.56± 9.98	121.15±6.79	145.45± 5.55
Platelets (×10^3^*μ*L^−1^)	1007± 116.62	2721.5± 28.86###	2419.75±72.64	1453.06± 84.64*∗∗∗*	1416.03± 10.69*∗∗∗*
MCH (pg)	7.5875± 0.66	7.36±0.13	6.95± 0.93	6.55± 0.68	6.1± 0.28
Hemoglobin (g/dL)	139.5± 6.20	143± 1.32	149± 3.31	137.25±3.00	154.75± 0.33
MCHC (g/dL)	2493± 222.73	1945± 35.79	3159.37± 122.81∗	2460.33± 324.42	2374±67.70

NOR-1: normal rats fed with SD treated with cotton oil; NOR-2: normal rats fed with HFHS treated with cotton oil; DMBA: negative control treated with cotton oil; TAM + DMBA: animals treated with tamoxifen (3.3 mg/kg); Neem + DMBA: animals treated with neem (*Azadirachta indica*) oil at a dose of 3 mL/kg at three different frequencies. All groups except the normal groups (NOR-1 and NOR-2) received subcutaneous dose of DMBA at the dose of 50 mg/kg. Data are represented as mean ± SEM (n = 5). #p < 0.05, ###p < 0.001 as compared to normal rats (NOR-2); *∗*p < 0.05, *∗∗∗*p < 0.001 as compared to DMBA.

## Data Availability

The data used to support the findings of this study are available from the corresponding author upon request.

## Abbreviations

AST: Aspartate transaminase
BW: Body weight
CC_50_: Cytotoxic concentration for 50% of the cells
DMBA: 7,12-dimethylbenz(a)anthracene
DMSO: Dimethylsulfoxide
ER: Estrogen receptor
Hb: Hemoglobin
Ht: Hematocrit
HFHS: High-fat/sucrose diet
NHC: National Herbarium of Cameroon
RBC: Red blood cell
SD: Standard diet
SEM: Standard error on mean.
